# The Association of Dietary Micronutrient Intake and Systemic Inflammation among Patients with Type 2 Diabetes: A Cross-Sectional Study

**DOI:** 10.3390/healthcare12181804

**Published:** 2024-09-10

**Authors:** Kenneth Izuora, Amalie Alver, Arpita Basu, Kavita Batra, Shelley J. Williams, Jeffrey L. Ebersole

**Affiliations:** 1 Department of Internal Medicine, Kirk Kerkorian School of Medicine at UNLV, University of Nevada, Las Vegas, NV 89102, USA; alver@unlv.nevada.edu; 2 Department of Kinesiology and Nutrition Sciences, School of Integrated Health Sciences, University of Nevada, Las Vegas, NV 89154, USA; arpita.basu@unlv.edu; 3 Department of Medical Education and Office of Research, Kirk Kerkorian School of Medicine at UNLV, University of Nevada, Las Vegas, NV 89102, USA; 4 School of Dental Medicine, University of Nevada, Las Vegas, NV 89102, USA; shelleyj.williams@unlv.edu (S.J.W.); jeffrey.ebersole@unlv.edu (J.L.E.)

**Keywords:** micronutrient, inflammation, type 2 diabetes

## Abstract

Inflammation contributes to the pathogenesis of type 2 diabetes (T2DM). This study sought to document how the systemic biomarkers of inflammation varied based on food choices among patients with T2DM. This cross-sectional study enrolled ambulatory patients with T2DM. Demographic and clinical information was collected. Five drops of fingerstick blood were collected using an absorbent paper device (HemaSpot HFR). C-reactive protein (CRP), serum amyloid A protein (SAA), and fibrinogen were measured using a Luminex assay. Patient-generated 7-day food diaries were analyzed using a validated food processor software. Data were analyzed by Pearson’s correlation tests, linear regression and logistic regression with the significance level set at 0.05. Among the 71 participants, 43 (60.6%) were females. The average age and duration of T2DM were 64.1 ± 10.3 and 15.8 ± 9.1 years, respectively. In a simple linear regression run with selected micronutrients, iron [F (1, 53) = 5.319, *p* < 0.05, adj. R^2^ = 0.074] significantly predicted plasma CRP. This significance was lost with multiple linear regressions including age, gender, BMI, T2DM duration, T2DM complications, glycohemoglobin A1c (HbA1c) and other micronutrients. The average intake of most micronutrients by the participants was below the recommended daily intake. A higher intake of iron-rich foods was associated with higher levels of systemic inflammation in a simple linear regression model, but the association was not present after adjusting for patient factors like age, gender, BMI and T2DM-related variables. This relationship needs to be explored further given the key role of inflammation in the pathogenesis of T2DM and its associated complications.

## 1. Introduction

Cardiovascular disease (CVD) is a leading cause of mortality worldwide [1,2]. Nutrition in general plays a pivotal role in the development, progression and management of cardiovascular disease [3,4]. There is abundant evidence to support the influence of nutritional choices on cardiovascular outcomes [5,6,7]. Inflammation is a major mechanism through which environmental factors, including nutrition, can impact health outcomes. Several studies have linked inflammation with adverse health outcomes like hypertension, dyslipidemia, worsening T2DM and CVD, among other metabolic disorders [8,9,10,11,12]. A key approach to reducing these adverse health outcomes is through reducing systemic inflammation.

Various foods can modulate the magnitude of systemic inflammation and hence contribute to the etiology of the associated adverse health outcomes [13,14,15,16,17]. As an example, the Mediterranean diet, that is rich in vegetables, fruits, nuts, cereals, and olive oil, has been associated with decreased inflammation [18,19,20]. Conversely, fried and processed foods containing lipid oxidation products have been linked to increased inflammation and adverse CVD health outcomes [21,22,23]. This dietary relationship is especially important among individuals who are at increased risk for adverse CVD outcomes such as patients with T2DM.

A significant challenge in assessing the impact of nutrition on health outcomes stems from the inherent complexity of food composition. Most foods contain a diverse array of macro- and micronutrients in varying amounts [24,25]. To better appreciate the association between health outcomes and diet, it is relevant to break down specific diets into their constituent nutritional elements. Although some correlations have been established between micronutrient intake and CVD, there is still a need to better understand the mechanisms underlying these associations [26,27].

The objective of this study was to evaluate the relationship between the micronutrient content of the diet of patients with T2DM and systemic biomarkers of inflammation to understand how variation in dietary micronutrients might contribute to modulating systemic inflammation.

## 2. Materials and Methods

### 2.1. Study Design and Setting

This cross-sectional study was conducted at the out-patient endocrinology clinic of the University of Nevada Las Vegas (UNLV). All potential participants were approached consecutively during their routine clinic visit for study participation. Following an explanation of the study’s procedures, informed consent was obtained from eligible participants. This study was approved and overseen by the UNLV Institutional Review Board (IRB number: 1414893-6).

### 2.2. Inclusion/Exclusion Criteria

Subjects included in the study were ambulatory adult patients with T2DM who were >18 years old. Each patient had an established diagnosis of T2DM over a duration of at least 1 year prior to study entry. Finally, subjects were required to be able to understand and give informed consent. Individuals were excluded if they had a diagnosis of T2DM for a duration of less than 1 year, a diagnosis of type 1 diabetes, or if they were unable to give informed consent.

### 2.3. Study Procedures

Main Study Questionnaire: The questionnaire was administered by a study team member to capture demographic information (age, gender, race/ethnicity); nutritional information to identify factors that impact healthy food choices (e.g., proximity to healthy food, mobility, financial limitations); health information related to T2DM (i.e., body mass index (BMI), T2DM duration); and the presence of CVD complications (microvascular and macrovascular).

Nutrition Assessment: Actual food intake was captured using a standard food diary. Patients were asked to record all their food and beverage intake for a one-week period immediately after their initial clinic visit. Participants were asked to document the amount of each food, recorded along with a detailed description of the food. They were provided with a self-addressed and stamped envelope, which they used to mail the food diary back to the study team. The diaries were reviewed by the study dietician (AB), and all food logged over the one-week entry period was analyzed by the study dietitian using food analysis software (ESHA Research, Salem, OR, USA) to estimate the average daily micronutrient intake. The ESHA food analysis software (version 11.1) contains a database with nutrition information for over 72,000 commonly consumed foods. Food analysis using the ESHA software has been validated to correlate strongly with the National Health and Nutrition Examination Survey (NHANES) food database [28].

Medical Assessment: As a part of their clinic visit, patients were routinely evaluated for management of their T2DM. The average HbA1c from the preceding 3 years was calculated to reflect the achievement of long-term glycemic goals. Information provided regarding T2DM history and complications and CVD status was verified through a review of past physical examinations and diagnostic procedures in the electronic health records.

### 2.4. Biomarker Assessment

Biomarker assessments were conducted at the research laboratory of the UNLV School of Dental Medicine (JE, SW). Using a 28-gauge lancet, 5 drops of finger-stick blood were collected using a HemaSpot^®^ HF (Spot on Sciences, San Francisco, CA, USA) cartridge for each participant. The cartridges were stored at −20 °C until being analyzed using Luminex^®^ technology and Life Technologies ProcartaPlex kits (Thermo Fisher Scientific, USA) for targeted biomarkers of inflammation (CRP, serum amyloid A protein, fibrinogen). For the Luminex assessment, blood was eluted from dried spots by transferring 1 cartridge and an absorbent paper blade into a 1.5 mL Eppendorf microcentrifuge tube with 100 µL of extraction buffer (0.01 M KH2PO4, pH 7.0). The eluent was vortexed for 30 min at ambient temperature and centrifuged for 5 min at 10,000× *g* to pellet the filter blade and particulates. Total protein was measured by Coomassie Bradford method (Thermo Fisher Scientific, USA) for normalization of the blood spot values.

### 2.5. Statistical Analysis

Univariate and bivariate tests were conducted. All assumptions, including normality and homogeneity of variance, were assessed. Categorical variables were represented as frequencies and proportions, whereas continuous variables were represented by mean and standard deviations. A Pearson’s product–moment correlation was run to assess the relationship between the different biomarkers and micronutrients. A simple linear regression was run followed by standard multiple regression tests to predict CRP from micronutrients, such as vitamins B6, B9, B12 and E and iron, in addition to age, gender, BMI, duration of diabetes in years, complications and HbA1c levels. The significance level was set at 0.05. A logistic regression was performed to ascertain the effects of age, gender, BMI, duration of diabetes in years, HbA1c and micronutrients on the likelihood that participants would have CRP above a value of 0.7 ng/mg, which was a cut-off point that was statistically determined by the Median Split Method. A model of logistic regression was fit to generate an adjusted odds ratio. Estimates for the parameters were obtained through the maximum likelihood estimation method with 95% Wald’s confidence limits for the logistic model. The final model was selected based upon the Akaike information criterion and The Schwarz Criterion [29]. The significance level was set at 0.05. The normal approximation to the binomial distribution method was used to calculate 95% confidence intervals of proportions in the univariate analyses. All analyses were conducted using SPSS version 27 or SAS 9.4.

## 3. Results

### 3.1. Descriptive Statistics

Among the 71 participants that met the inclusion criteria, 43 (60.6%) were female and 26 (36.6%) were white. The mean age and duration of T2DM were 64.1 ± 10.3 and 15 ± 9.1 years, respectively. The mean BMI of the population was 34.0 ± 8.3 kg/m^2^, with over 65% of the participants being obese (BMI ≥ 30 kg/m^2^). Nearly 60% of the participants had microvascular complications and about a quarter had macrovascular complications of diabetes (Table 1).

The recorded average intake of micronutrients by the participants, shown in Table 2, was consistently below the recommended dietary allowances (RDAs) for all micronutrients for the female population and, except for B6, B12 and iron, were also below the RDA for the male population.

### 3.2. Bivariate Statistics

As shown in Table 3, Pearson’s correlation coefficients between biomarkers and micronutrients showed that CRP was positively and moderately correlated with the SAA (R^2^ = 0.678, *p* < 0.001), and weakly correlated with fibrinogen (R^2^ = 0.289, *p* < 0.05) and iron (R^2^ = 0.302, *p* < 0.001).

### 3.3. Regression Analysis

In the simple linear regression run individually with micronutrients (vitamins B6, B9, B12 and E and iron), only iron [F (1, 53) = 5.319, *p* < 0.05, adj. R^2^ = 0.074] significantly predicted CRP levels. However, the multiple regression model, that included selected variables related to inflammation (age, gender, BMI, T2DM duration, T2DM complications, A1c and other micronutrients), did not predict CRP, in that F statistics (11, 43) = 1.567, *p* > 0.05, adj. R^2^ = 0.104. Regression coefficients and standard errors of the multiple regression are shown in Table 4.

### 3.4. Logistic Regression Analysis for Micronutrients

For the logistic regression, the probability was modeled on CRP > 0.7 ng/mg = yes, which constituted n = 32 (45.1%) of the sample. The cut-off of CRP above 0.7 ng/mg was statistically determined by the Median Split Method. Of the 11 predictor variables for CRP, that included age, gender, BMI, T2DM duration, HbA1c, presence of complications, and micronutrients, only BMI and HbA1c were significant predictors for CRP more than 0.7 ng/mg. In other words, with each increase in unit of BMI, the odds of CRP level above 0.7 increased by 1.23 times. Also, with each increase in unit of HbA1c, the odds of CRP level above 0.7 increased by 2.34 times (Table 5).

## 4. Discussion

In this study examining the relationship between dietary nutrients and presence of systemic inflammation among a cohort of patients with established T2DM, we observed significant correlations between the three biomarkers of inflammation (CRP, serum amyloid a protein, fibrinogen) consistent with previous reports [30,31,32]. There are multiple plausible biological mechanisms to explain the association between dietary micronutrients and inflammation. Many micronutrients, such as vitamins C and E, selenium and zinc, influence via their antioxidant defense systems by neutralizing free radicals and reactive oxygen to reduce oxidative stress and subsequent inflammation [33]. Additionally, vitamin D helps in regulating immune responses [34]. Zinc modulates inflammatory pathways and is vital for the proper functioning of enzymes [35]. Micronutrients can affect the cell signaling and gene expression related to inflammation via inflammatory and anti-inflammatory mediators [36,37,38]. For instance, the integrity of the gut lining and prevention of systemic inflammation is largely influenced by vitamin A and zinc [36,37,38]. This underscores the need to achieve a proper balance and interaction between micronutrients to reduce inflammation and chronic diseases [36,37,38].

In our study, a higher intake of iron-rich food was associated with higher levels of CRP, as was found in the simple regression. This relationship was not present in multiple regression models, where only the patient’s BMI predicted inflammation. No significant relationships between the intake of other micronutrients, including vitamin E, vitamin B6, vitamin B12 or B9, and the systemic inflammation measured by the CRP was noted. As was expected, higher BMI and higher HbA1c predicted CRP levels above the median for this population.

The intake of various micronutrients has been reported to affect systemic inflammation. For example, foods rich in choline, folic acid, vitamin E and zinc have been associated with lower levels of inflammation and CRP [39,40,41,42]. Contrary to these reports, our study did not find a significant reduction in inflammation related to any of these micronutrients. A possible explanation for the lack of association between these micronutrients and inflammation/CRP in this study may be due to the low consumption of these dietary factors by the majority of our study participants. The average daily consumption of choline, folate, vitamin E and zinc documented by our participants was much lower than the federal RDA guidelines for these micronutrients. In contrast to previously published studies where these micronutrients were specifically supplemented to optimize their intake, the participants in this study were requested to maintain their typical dietary patterns during the one week of dietary data collection.

Iron is an essential micronutrient for red blood cells and muscle oxygen transport. However, iron has also been reported to have deleterious health effects, especially through increasing systemic inflammation. Although adequate iron intake is necessary for normal physiologic function and cellular metabolism, when taken in large amounts, iron can mediate oxidative stress and inflammation, resulting in adverse health outcomes [43,44,45]. Hence, it is important to maintain an appropriate balance between iron intake and iron needs to maintain iron homeostasis in the body. The association between high dietary iron intake and higher CRP levels among our study participants is consistent with these reports. A high intake of food rich in iron, such as red or processed meats, has been implicated in the pathogenesis of adverse cardiometabolic outcomes [46,47,48]. The average iron intake in our population was above the RDA for males and below the RDA for females. An individual review of the food logs of our study participants with the highest iron levels (all above the RDA for iron) confirmed a high intake of red and processed meats, including meatloaf, spam, smoked sausage, bacon, pepperoni and steak. Multivariate regression models did not identify a significant relationship between iron intake and CRP. This may reflect the relationship between iron and the other variables in the model, like age, gender, BMI and other micronutrients, which may influence the relationship between the consumption of iron-rich foods and CRP.

A limitation of the study is the potential recall bias in the self-reported food diaries. This was addressed by asking the participants to promptly document their diet in the diary during or shortly after a meal. However, a strength of the approach is the average number of days of food records documented in the 7-day food diaries by each participant, which should provide a more comprehensive reflection of their average daily food intake. Also, because of the observational nature of this study, the findings are more likely to capture habitual dietary patterns that reflect real-life dietary choices; hence, our study is potentially more generalizable than an interventional approach. Another limitation is the relatively small sample size of the population, which may not adequately represent robust diversity in dietary intake across the population. A larger study will be needed to address this limitation. Finally, our method for measuring the biomarkers was not through a standardized CLIA laboratory-based assay. The study explored a sample collection and analytic approach that could be adopted in regions or populations with more limited access to professional medical care and laboratory availability. The correlations between the three biomarkers with similar mean and median values for CRP suggested a normal distribution, and that the assay method had some reasonable precision in reflecting variations in the biomarkers within this patient sample.

## 5. Conclusions

The overall implication of the study findings is that the inadequate consumption of foods rich in healthy micronutrients continues to represent a missed opportunity to intervene through the diet to improve cardiometabolic risk in T2DM patients. On the other hand, the consumption of diets containing iron-rich foods may contribute to the systemic pro-inflammatory state that could predispose individuals to adverse cardiometabolic outcomes. However, this relationship was modulated by patient factors like age, gender, BMI and T2DM-related variables and needs to be explored further.

## Figures and Tables

**Table 1:** Descriptive statistics of study population (N = 71).

Variable	Characteristics	Mean ± SD	n (%)	95% CI (LCL, UCL)
Age (years)	-	64.10 ± 10.3	-	61.6, 66.5
BMI (kg/m^2^)	-	34.0 ± 8.3	-	32.0, 36.0
BMI Status	Normal (18.5–24.9)	-	11 (15.5)	8.0, 26.0
	Overweight (25.0–29.9)	-	12 (16.9)	9.1, 27.6
	Obese (≥30.0)	-	48 (67.6)	55.5, 78.2
Gender	Female	-	43 (60.6)	48.3, 72.0
	Male	-	28 (39.4)	28.0, 51.8
Race/ethnicity	White	-	26 (36.6)	25.5, 48.9
	Non-white	-	45 (63.4)	51.1, 74.5
Duration of T2DM (years)		15.80 ± 9.1	-	13.7, 18.0
Average A1c over last 3 years (%)	-	7.80 ± 1.5	-	7.5, 8.2
CRP (ng/mg)	-	0.74 ± 0.19	-	0.70, 0.80
Microvascular complications	Yes	-	42 (59.2)	46.8, 70.7
	No	-	29 (40.8)	29.3, 53.2
Macrovascular complications	Yes	-	16 (22.5)	13.5, 34.0
	No	-	55 (77.5)	66.0, 86.5

Note: The percentages of the categories may not be equal to 100% because of unreported values.

**Table 2:** The mean micronutrient intake of participants (N = 71).

Micronutrient	Mean ± SD	Mean ± SD (Males)	Mean ± SD (Females)	RDA * (Male)	RDA (Female)
Vitamin E (mg)	4.27 ± 3.83	4.71 ± 5.23	3.97 ± 2.59	11	15
Vitamin B6 (mg)	0.95 ± 0.47	1.15 ± 0.62	0.81 ± 0.29	1	1.2
Vitamin B9 (mcg)	245.3 ± 163.63	287.68 ± 226.40	216.96 ± 99.05	400	400
Vitamin B12 (mcg)	2.50 ± 1.58	3.20 ± 2.09	1.97 ± 0.87	1.8	2.4
Zinc (mg)	5.42 ± 2.90	6.34 ± 3.58	4.84 ± 2.22	8	9
Iron (mg)	9.75 ± 4.51	11.31 ± 5.68	8.72 ± 3.24	8	18
Choline (mg)	173.02 ± 87.64	198.96 ± 92.85	155.99 ± 81.03	375	400

* RDA: recommended dietary allowances; SD = standard deviation.

**Table 3:** Pearson’s correlations between biomarkers and micronutrients.

Variables	CRP	Fibrinogen	SAA	Vitamin E	Vitamin B6	Folate	Vitamin B12	Iron
CRP	1	0.289 *	0.678 **	0.068	0.222	0.207	0.125	0.302 **
Fibrinogen	0.289 *	1	0.301 *	0.061	0.125	0.140	0.088	0.180
SAA	0.678 **	0.301 *	1	0.174	0.194	0.118	0.048	0.216
Vitamin E	0.068	0.061	0.174	1	0.218	0.181	−0.019	0.273 *
Vitamin B6	0.222	0.125	0.194	0.218	1	0.828 **	0.882 **	0.863 **
Vitamin B9	0.207	0.140	0.118	0.181	0.828 **	1	0.769 **	0.877 **
Vitamin B12	0.125	0.088	0.048	−0.019	0.882 **	0.769 **	1	0.759 **
Iron	0.302 *	0.180	0.216	0.273 *	0.863 **	0.877 **	0.759 **	1

** *p* < 0.01; * *p* < 0.05.

**Table 4:** Multiple linear regressions predicting CRP by selected independent variables.

Variable	Unstandardized Coefficient	Standardized Coefficient Beta	Coefficients Standard Error	95% CI (LCL, UCL)		*p* Value
Constant	−2.284	-	1.851	−6.017	1.449	0.2
Age	0.001	0.002	0.019	−0.038	0.039	0.9
Gender (Ref: Females)	0.138	0.056	0.388	−0.645	0.920	0.7
Body mass index	0.047	0.303	0.024	−0.001	0.096	0.06
Duration of diabetes (years)	−0.014	−0.106	0.022	−0.058	0.030	0.5
HbA1c (%)	0.119	0.134	0.130	−0.143	0.382	0.4
Complications (Ref: No)	0.206	0.081	0.369	−0.538	0.950	0.6
Vitamin E	0.002	0.006	0.053	−0.106	0.110	0.9
Vitamin B6	0	0.204	1.012	−1.514	2.569	0.6
Vitamin B9	−0.002	−0.315	0.002	−0.007	0.002	0.3
Vitamin B12	−0.188	−0.248	0.252	−0.696	0.320	0.5
Iron	0.139	0.499	0.092	−0.047	0.324	0.3

**Table 5:** Logistic regression analysis predicting the likelihood of CRP over 0.7 based on selected independent variables, including micronutrients (N = 71).

Variable	Adjusted Odds Ratio	95% CI (LCL, UCL)		*p* Value
Age	0.993	0.916	1.077	0.9
Gender (Ref: Females)	4.117	0.665	25.505	0.1
Body mass index	1.232	1.063	1.426	0.005
Duration of diabetes (in years)	1.052	0.949	1.167	0.3
HbA1c	2.349	1.123	4.912	0.023
Complications (Ref: No)	0.312	0.050	1.959	0.2
Vitamin E	1.150	0.894	1.478	0.3
Vitamin B6	0.078	0.001	6.757	0.2
Vitamin B9	0.990	0.978	1.001	0.08
Vitamin B12	1.819	0.605	5.464	0.3
Iron	1.250	0.855	1.827	0.2

Probability was modeled on CRP > 0.7 = yes, which constituted n = 32 (45.1%) of the sample.

## Data Availability

The datasets presented in this article are not readily available due to ethical reasons.
